# Risk Factors Associated with Mortality in Hospitalized Patients with COVID-19 during the Omicron Wave in Brazil

**DOI:** 10.3390/bioengineering9100584

**Published:** 2022-10-20

**Authors:** Marilaine Colnago, Giovana A. Benvenuto, Wallace Casaca, Rogério G. Negri, Eder G. Fernandes, José A. Cuminato

**Affiliations:** 1 Institute of Chemistry, São Paulo State University (UNESP), Araraquara 14800-060, Brazil; 2 Faculty of Science and Technology (FCT), São Paulo State University (UNESP), Presidente Prudente 19060-900, Brazil; 3 Institute of Biosciences, Letters and Exact Sciences, São Paulo State University (UNESP), São José do Rio Preto 15054-000, Brazil; 4 Science and Technology Institute, São Paulo State University (UNESP), São José dos Campos 12247-004, Brazil; 5 Immunization Division—Centre of Epidemiology Surveillance of the São Paulo State Health Department, São Paulo 01246-000, Brazil; 6 Institute of Mathematics and Computer Science, São Paulo University (USP), São Carlos 13566-590, Brazil

**Keywords:** COVID-19, mortality, risk factors, hospitalization, Omicron

## Abstract

Considering the imminence of new SARS-CoV-2 variants and COVID-19 vaccine availability, it is essential to understand the impact of the disease on the most vulnerable groups and those at risk of death from the disease. To this end, the odds ratio (OR) for mortality and hospitalization was calculated for different groups of patients by applying an adjusted logistic regression model based on the following variables of interest: gender, booster vaccination, age group, and comorbidity occurrence. A massive number of data were extracted and compiled from official Brazilian government resources, which include all reported cases of hospitalizations and deaths associated with severe acute respiratory syndrome coronavirus 2 (SARS-CoV-2) in Brazil during the “wave” of the Omicron variant (BA.1 substrain). Males (1.242; 95% CI 1.196–1.290) aged 60–79 (3.348; 95% CI 3.050–3.674) and 80 years or older (5.453; 95% CI 4.966–5.989), and hospitalized patients with comorbidities (1.418; 95% CI 1.355–1.483), were more likely to die. There was a reduction in the risk of death (0.907; 95% CI 0.866–0.951) among patients who had received the third dose of the anti-SARS-CoV-2 vaccine (booster). Additionally, this big data investigation has found statistical evidence that vaccination can support mitigation plans concerning the current scenario of COVID-19 in Brazil since the Omicron variant and its substrains are now prevalent across the entire country.

## 1. Introduction

COVID-19 is a respiratory infection caused by the SARS-CoV-2 virus, popularly known as the novel coronavirus. The first case of the disease in Brazil was reported on 26 February 2020, in the city of São Paulo [1]. Due to high transmissibility, on 11 March 2020, the World Health Organization (WHO) declared the novel coronavirus (COVID-19) outbreak a global pandemic, with a significant possibility of the disease leading to high rates of morbidity and mortality [1].

The SARS-CoV-2 pandemic has led to a considerable social, economic, and humanitarian impact, especially in developing countries [2,3], such as Brazil, which has experienced recurrent infection waves caused by the emergence of new COVID-19 variants. Among the strains of greatest concern, we can cite Omicron (B.1.1.529), which was detected in November 2021 in Botswana and South Africa, leading to a rapid spread of the disease on a global scale [4]. Indeed, the Omicron variant contains an expressive number of spike protein mutations [5], which may justify the immune-escape response and the high capability for transmission and reinfection, so that it may be up to 10 times more contagious than the original COVID-19 strain and two times higher than the Delta variant [6,7,8].

Considering the most important pharmaceutical interventions to mitigate the number of hospitalizations and deaths from Omicron, we can highlight the third dose (booster) of the anti-COVID-19 vaccine, which is capable of considerably reducing the most severe cases of the disease while still attenuating the virus transmission [9]. In Brazil, the disease containment strategy has focused on immunization campaigns to increase immunity against Omicron. On 20 December 2021, the Brazilian Ministry of Health announced the availability of booster doses for individuals over 18 years of age, as well as the possibility of providing an additional immunization shot for the group of immunocompromised individuals [10]. As vaccination progresses, it becomes more important to identify those at greatest risk of post-vaccination infection, ensuring that individuals at relatively high levels of risk will be protected, based on both non-/pharmaceutical interventions, including vaccination campaigns and basic lifestyle precautions [11].

Another critical aspect that significantly influences the number of COVID-19 hospitalizations and deaths are risk factors such as age, gender, and the presence of comorbidities. Although previous studies showed that some risk factors such as comorbidities [12,13] and advanced age [14,15] can increase the risk of severity and mortality in patients with COVID-19, most existing studies were conducted in the pre-Omicron period, whose analyses and goal focused on investigating the impact of other variants of concern such as Alpha [16] and Delta [17].

Moreover, among the recent papers specifically devoted to coping with risk factors for hospitalized patients during the Omicron period, the majority of proposals aim to explore the data as a case study with a limited number of patients from a given hospital or medical center [18,19]. Indeed, only a few studies were proposed in the post-Omicron period to properly measure and quantitatively assess the risk factors in entire countries [20,21,22], especially for developing countries such as Brazil. Concerning this particular category of works, Sheikh et al. [20] assessed the severity of the Omicron variant, investigating how booster shots of the vaccines can be effective in preventing symptomatic infections. The authors collected nationwide data from the Scotland-wide Early Pandemic Evaluation and Enhanced Surveillance (EAVE II) platform to obtain initial estimates of COVID-19 severity with Omicron, as well as the effectiveness of additional doses for symptomatic infection from the Omicron variant. In a similar work, Bager et al. [21] analyzed the risk of hospitalization related to Omicron against Delta variant in Denmark, a nation with significant testing capability and a high immunization coverage rate. In their study, they found that the vaccination was associated with a lower risk of hospitalization compared to individuals with no doses or only one shot of vaccine, and a significantly lower rate of hospital admission with Omicron compared to Delta cases. Similar findings were also reached by Butt el al. [22] when evaluating the National COVID-19 Dataset in Qatar, where among the individuals infected with Omicron, 0.03% had several/critical disease, compared to 1.5% with Delta. Factors associated with less severe disease included vaccination with booster doses.

In order to advance the literature dedicated to the Omicron variant while assessing its severity degree in a very large population of hospitalized patients with COVID-19, this study aims to identify, measure, and statistically verify different risk factors for death among inpatients during the Omicron wave in Brazil. In contrast to most existing studies that address earlier SARS-CoV-2 variants such as Alpha and Delta strains, the conducted research focused on investigating the most persistent risk factors in people admitted to Brazilian hospitals during the wave of the Omicron variant (BA.1 substrain) from a nationwide perspective, i.e., by collecting and analyzing a massive number of data extracted from official Brazilian government resources, which include all reported cases of hospitalizations and deaths associated with SARS-CoV-2 in Brazil.

More specifically, a big data-driven analysis was performed to quantitatively assess all clinical cases of the Omicron variant in Brazil concerning the following risk factors: gender, age, vaccination, and commodities. Such knowledge allows for a better understanding and categorization of what groups of individuals are more vulnerable to acquiring more severe manifestations of the disease. As a result, public authorities can take more effective actions such as the implementation of massive vaccination campaigns in order to prioritize less resilient groups of people while ensuring longer-term protection. Another important contribution of this study concerns the assessment of the vaccine booster shot in the post-Omicron context. Our results indicate that the booster dose substantially contributes to lowering the risk of death.

## 2. Materials and Methods

### 2.1. Data Source

In this study, SARS hospitalization and death records were gathered from the Brazilian hospitalization database operated by the Ministry of Health—the Influenza Surveillance System Data Repository [23] (SIVEP-Gripe, in Portuguese), which monitors SARS hospitalization cases in the whole country, including those of COVID-19 [24,25,26]. In our data evaluation, we focused our analysis only on hospitalized patients with SARS whose final classification for the cases was caused by the novel SARS-CoV-2 coronavirus. The database was acquired on 25 April 2022 at https://opendatasus.saude.gov.br/dataset/srag-2021-e-2022, and it comprises records collected throughout Brazil.

### 2.2. Inclusion Criteria

The variables from the SIVEP-Gripe database considered for analysis were as follows: (i) gender, (ii) booster vaccination, (iii) age group, and (iv) comorbidities. The gender variable was divided into two categories: female (F) and male (M). The latter was considered a risk factor for the sake of comparison [27,28]. Regarding the age group, patients over 18 years of age, hospitalized during the Omicron wave in Brazil between 1 February and 31 March 2022, were included. In our analysis, hospitalized individuals aged 40–59, 60–79 years, and 80 years or above were considered a risk group. Concerning the investigated comorbidities, the categorical variable (yes or no) from the SIVEP-Gripe database was taken to indicate the presence of comorbidities. In more technical terms: if the answer for this variable is yes, a new set of categories is created to specify what the comorbidities of each case were. The Brazilian Ministry of Health system presents twelve comorbidities already entered, namely, (i) postpartum women, (ii) cardiovascular disease, (iii) hematologic disease, (iv) Down syndrome, (v) liver disease, (vi) asthma, (vii) diabetes, (viii) neurological disease, (ix) pneumopathy, (x) immunodeficiency or immunocompromised, (xi) kidney disease, and (xii) obesity [23]. If a certain patient has any comorbidity in addition to those listed above, the system allows the user to enter this manually.

Regarding vaccination, the SIVEP-Gripe database is integrated with the Brazilian Immunization Program [29]. Particularly, a form is automatically filled in if a certain patient has been vaccinated, as well as the immunization dates and other complementary information about each dose received. As a result, in this study, the hospitalization cases where the field related to the booster shot date was indicated as completed were classified as vaccinated patients.

In summary, the inclusion criteria adopted for this study were as follows:

- Population: inpatients over 18 years old, obtained from the SIVEP-Gripe database records [23], testing positive for COVID-19.
- Risk factors: gender (M), age (40–59 years; 60–79 years; and 80 years or older), incomplete vaccination (i.e., without the third dose of vaccine), and comorbidities (as previously listed).
- Analysis period: 1 February to 31 March 2022.

### 2.3. Exclusion Criteria

In order to adequately deal with missing data and incorrectly filled fields, we follow [30,31] so that records containing inconsistent data such as negative ages; records containing a blank field in the gender variable, records whose patient was vaccinated but without the vaccination date field filled in, and records containing missing data were excluded from the collected data.

### 2.4. Statistical Analysis

Descriptive statistical analyses were performed by using absolute (*n*) and relative (%) frequencies. The fatality rate was also computed, which comprises the calculation of the proportion between deaths and the total number of people hospitalized due to COVID-19 in each analyzed group.

In our quantitative assessments, the odds ratio (OR) was also gauged to determine the increased risk of death from COVID-19 in the following cases:

- Hospitalized patients aged 40–59 years, aged 60–79 years, and 80 years or older.
- Male hospitalized patients.
- Those with at least one comorbid condition.
- Those with an incomplete vaccination status.

The OR is an important statistical metric that is used to drive several epidemiology studies because it aims to measure the increased risk of incurring a certain disease if a particular factor is present [29].

Aiming at assessing the gathered data while identifying the factors that are most related to death, the logistic regression analysis was applied so that the OR values were statistically adjusted (with a 95% confidence interval) [32,33]. Before performing the regression analysis, a correlation study between the variables was conducted to eliminate the possibility of correlated explanatory variables (see [34] for implementation details).

### 2.5. Computing Platform and Programming Language

Data analysis was performed using the Google Colab platform, which allows users to write and execute arbitrary Python codes through the browser. Regarding the logistic regression analysis, the computational library statsmodels [35] was taken. Such a Python library provides several methods and programming functions for building statistical models as well as performing data analysis.

## 3. Results

From 1 February to 31 March 2022, 50,896 patients with COVID-19 were admitted to Brazilian hospitals according to the SIVEP-Gripe database. The patients were mainly male (51.85%), with a mean age of 68.71 years and a dispersion between 50.57 and 86.85 years, according to ±1 standard deviation (SD). Most of the individuals among the hospitalized patients (73.35%), and among the deaths (84.22%), were 60 years and over. Most of the inpatients (58.72%) had at least one comorbidity, and the minority (20.12%) had taken the COVID booster dose.

Among the hospitalized patients, 17,640 (34.66%) died, with an average fatality of 0.35. The fatality was higher among male patients (0.37) compared to female ones (0.32); patients aged over 80 years (0.46) compared to the following age groups: 18–39 years (0.13), 40–59 years (0.25), and 60–79 years (0.35); and patients with one (0.36), two (0.40), three (0.45), or four or more (0.48) comorbidities compared with the group of individuals without comorbidities (0.29). Regarding the booster dose, the fatality of the vaccinated group (0.36) was slightly higher than that of the non-vaccinated group (0.34); however, it should be noted that the first group totaled 21.08% of deaths, while the second one reached 78.98%. The statistics summary of the patients is given in Table 1.

**Figure 1:**
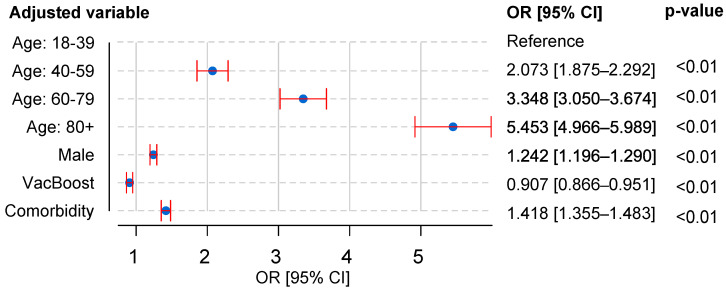
Adjusted odds ratio (OR) and 95% CI (red lines) for risk factors associated with COVID-19 mortality.

Figure 1 shows the adjusted OR values, indicating that there was a higher risk associated with some particular factors, where advanced age was the greatest risk factor (60–79 years old: OR 3.348; 95% CI 3.050–3.674; over 80 years old: OR 5.453; 95% CI 4.966–5.989). Males (1.242; 95% CI 1.196–1.290) or those individuals who had one or more comorbidities (1.418; 95% CI 1.355–1.483) were also considered as potential risk factors that influenced the chance of death. Concerning vaccinated inpatients, the adjusted OR value (0.907; 95% CI 0.866–0.951) reveals a reduction in the chance of dying if the patient received the booster shot of the anti-COVID-19 vaccine.

The adjusted logistic regression model demonstrated good calibration performance (*p*-value > 0.05) for the Hosmer–Lemeshow test [36]. A non-significant Hosmer–Lemeshow test (*p*-value > 0.05) indicates good calibration, and a significant test (*p*-value < 0.05) leads to poor calibration [36,37].

Advanced age and comorbidities in individuals hospitalized with COVID-19 not only impact deaths but also affect the most serious cases of the disease, such as those that require the intensive care unit (ICU). Regarding age, 35.9% of the total number of patients over 60 years of age were admitted to the ICU, compared to 31.6% of patients between 18 and 59 years. Considering the percentage of inpatients who died after being admitted to the ICU, 21% of occurrences were observed among elderly patients, compared to only 13% among younger hospitalized individuals.

**Figure 2:**
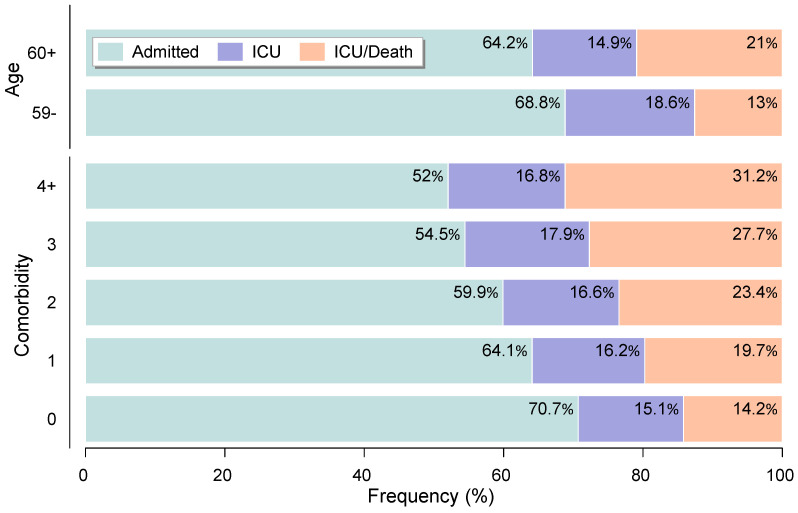
COVID-19 hospitalizations, intensive care unit (ICU) admissions, and deaths, by age groups and comorbidities.

Regarding chronic diseases, patients with multiple comorbidities had the highest percentages of ICU admission, followed by death. Almost half (48%) of the inpatients with four or more comorbidities required being admitted to ICU, while among hospitalized people without comorbidities, this value was below 30% (more details can be seen in Figure 2). Regarding deaths from ICU patients, the percentages were 14.2%, 19.7%, 23.4%, 27.7%, and 31.2% for hospitalized patients with none, one, two, three, and four or more comorbidities, respectively.

## 4. Discussion

It was found that the risk factor of being elderly (40–59, 60–79 or 80+ years old) increases the possibility of death compared to hospitalized adults under 40 years of age. This finding is in line with those reported in other studies conducted for different countries [14,38,39,40], and it can be interpreted due to the influence of immunosenescence (immune aging), which negatively contributes to a low immune response to vaccination of the elderly. Notice that this immunological fact is not unique to anti-COVID-19 vaccines, occurring with influenza, pneumonia, tetanus, and hepatitis B vaccines as well [41]. In addition, there is also reduced immunological memory generation in individuals with advantageous age, as well as the loss of antibodies, making this particular group more susceptible to infection [41]. The above-discussed issues together with the results presented in Section 3 further reinforce the importance of periodic immunization campaigns prioritizing specific groups of people, such as the elderly population.

Patients with comorbid conditions are highly correlated to a higher risk of death among hospitalized individuals (OR 1.418; 95% CI 1.355–1.483), and the consequences are more severe if the patient has multiple comorbidities [42,43]. Other studies in the specialized literature point out diabetes, hypertension, cardiovascular diseases, and obesity [44,45,46] as the comorbidities with the highest risk of mortality from COVID-19. Indeed, there are many specific factors that may explain the elevated risk of death among individuals with comorbidities. Patients with diabetes, for example, have alterations in phagocytic cells, which are essential for fighting inflammation, making it difficult to deal with infections [47]. Obesity (BMI ≥ 30 kg/m²), which is another risk factor related to the increased severity of influenza A (H1N1) [48], is associated with a decrease in expiratory reserve volume, functional capacity, and respiratory system compliance [49]. Concerning chronic diseases, such as those derived from cardiovascular and endocrine issues, it has been reported that hospitalized patients with such conditions are more susceptible to contracting and developing more severe cases of COVID-19, especially due to treatments carried out by these patients who react involuntarily on Angiotensin-converting enzyme 2 (ACE2), as SARS-CoV-2 infects the body when the spike protein binds to this human protein [50,51].

Although the results indicate that there is a higher probability of death in patients with chronic diseases when inspecting the scenario dominated by the Omicron variant and its substrains, the massive vaccination campaign with additional booster doses in Brazil has not targeted patients with comorbid conditions, i.e., only immunocompromised patients and age ranges were given top priority [52]. Therefore, more in-depth targeted studies are recommended to verify the need to prioritize this susceptible group of individuals when roll-outing anti-COVID-19 massive vaccination.

As shown in Figure 1, the OR score obtained for hospitalized male patients shows that they have a higher mortality rate than female patients (1.242; 95% CI 1.196–1.29). Other studies corroborate this finding [27,28,46] when investigating other SARS-CoV-2 variants such as Alpha, Gamma, and Delta, pointing out that male patients may have a greater expression of the ACE2 enzyme, which is regulated by male sex hormones, making this group of people more susceptible to infections and more severe SARS-CoV-2 cases [28,50]. In addition, women may have the advantage of having an immune system that detects pathogens earlier, as well as having higher immune responses than men, which can be explained by the high density of immune-related genes on the X chromosome [53].

Regarding the vaccine booster dose results, the majority (78.92%) of hospitalized patients who died had not been vaccinated with the booster dose. Although fatality among those who were vaccinated was slightly higher in terms of intra-group proportion, the adjusted OR value (0.907; 95% CI 0.866–0.951) indicates a lower risk of death in patients artificially immunized with the booster dose. It is important to note that, in contrast to other studies devoted to verifying the effectiveness of the vaccination for the Omicron variant, which mostly report that vaccine doses ensure additional protection in susceptible individuals, inducing more effective immune responses against symptomatic infection and hospitalization for COVID-19 [20,54,55,56], the present study focused on investigating and quantitatively measuring the effect of an extra vaccine shot concerning the mortality of hospitalized patients in Brazil. In light of this, the obtained assessments indicate the need for future clinical studies on the subject.

Considering the overall vaccination performance, a few studies reported that vaccine efficacy is lower with Omicron compared to other variants, advocating that the third shot of anti-COVID-19 vaccine is necessary to protect against symptomatic Omicron infection and serious outcomes [55,56,57]. Furthermore, as discussed by Andrews et al. [58], in South African, German, and British studies, they found a reduction in neutralizing activity of Omicron compared to neutralizing against the Delta variant [58]. The authors argue that cellular immunity also plays an important role in protecting against SARS-CoV-2 variants, even more than antibodies, as these decrease with time since infection or vaccination. Moreover, future research should be focused on reducing the production of new virus particles in the blood, thus acting as an inhibitor of virus replication, especially at the early stage with low virus load [59].

Our outcomes are consistent with recent studies carried out in other countries. The study presented by Lo et al. [60] applied multivariate logistic regression to estimate the risk of a serious outcome (hospitalization, ICU admission, or death) contrasted with mild COVID-19 cases among residents in the Alberta province (Canada). The adjusted odds ratios were highest for the 80+ age group (29.77; 95% CI 21.63–40.98), males (1.44; 95% CI 1.30–1.60), unvaccinated (16.1; 95% CI 13.8–18.8), and patients with three or more comorbidities (13.1; 95% CI 10.9–15.8). In a similar report, Jassat et al. [61] took nationwide data from the South Africa’s National Hospital Surveillance System to gauge the risk factors associated with mortality among SARS-CoV-2 hospitalized patients during the combined period of Delta, Omicron BA.1/BA.2, and Omicron BA.4/BA.5 waves. The odds ratio scores indicate the 60+ age (17.9; 95% CI 16.2–19.8), males (1.3; 95% CI 1.3–1.3), and presence of comorbidities (1.5; 95% CI 1.4–1.5) as potential risk factors. Finally, Nafilyan et al. [62] collected integrated data from the Office for National Statistics Public Health Data Asset (England) to infer the risk factors for COVID-19 death after receiving a booster dose of vaccine during the Omicron wave. The mortality risk was still more strongly correlated with age than with other factors, such as being male and having comorbidities. Notice that although the above-discussed studies differ from our investigation in terms of baseline to compute the OR scores as well as the type of COVID-19 variant, they all lead to the same conclusion concerning the risk factors. These include high OR values associated with advanced age, followed by the presence of comorbidities and being male. Vaccination also plays an important role in reducing more severe cases.

Despite the compatibility of our results with other works and the statistical validation performed on a massively scalable database at a nationwide level, the present study also has some issues that must be observed. First, since the analyzed variables were fully extracted from an electronic database, it becomes impractical to obtain a detailed clinical evolution of the more than 50,000 patients amidst the more than 10 million cells in the massive database [24,25]. Second, the national-level SIVEP-GRIPE database only gathers hospitalization SARS cases, i.e., it does not comprise individuals who tested positive for COVID-19 and were not hospitalized. According to the Brazil Civil Registry Office [63], 1227 COVID-19 deaths occurred outside of hospitals against 17,640, as listed in Table 1 during the study period. Third, not exactly all COVID-19-related deaths may be reported in the SIVEP-Gripe repository, such as out-of-hospital deaths without any testing for confirmation of SARS-CoV-2. All these issues lead to another scope of evaluation in the future since they depend on the integration and availability of public data by the Brazilian government. Lastly, Brazil is a middle-income nation with historic social inequalities that may impact the quality of regional healthcare services, including poor clinical assistance. For example, from Table 1, among 17,640 patients who died, 5757 (32.6%) were not admitted to the ICU, likely due to a lack of access to appropriate hospital beds, thus reflecting regional economic disparities even within the same region. Despite the influence of socioeconomic factors on mortality, such as the location of residence, human development index, distance to the hospitals, and the level of education [64], in our study, these were not considered as many of them are hard to measure and correlate in a large multicultural country such as Brazil.

## 5. Conclusions

This study statistically verified that advanced age, the presence of comorbidities, and being male increased mortality in hospitalized patients with SARS-CoV-2 during the wave of COVID-19 emerged by the Omicron variant (BA.1) in Brazil. In particular, among the risk factors studied, it was found that individuals who were over 60 years of age had the greatest impact on the increased risk of death from the disease. The study also revealed that the anti-COVID-19 booster vaccine dose contributed to a decrease in the probability of death. Considering this finding for Omicron cases in Brazil, which is in line with other studies in the specialized literature [14,20,46,55], this research reinforces the adoption of massive immunization strategies that aim to prioritize groups of individuals that are more vulnerable to acquiring more severe conditions of the disease, promoting the application of booster doses while implementing periodic vaccination campaigns.

It is also worth mentioning that since the Omicron variant and its substrains are relatively new, there are a lack of concrete data and research surveys concerning the impact of Omicron, especially at the nationwide level. In fact, most of the existing studies [16,17,65] address the advancement of previous SARS-CoV-2 variants, such as the original strain, Alpha, Delta and Gamma. In contrast, our investigation focused on assessing the consequences of Omicron in Brazil, a multicultural country of vast territorial extension.

## Figures and Tables

**Table 1:** Personal and medical statistics for confirmed SARS-CoV-2 hospital admissions between 1 February and 31 March 2022, in Brazil.

Characteristics	COVID-19 Hospitalizations		COVID-19 Deaths		Lethality
	($n_{h}$ = 50,896)		($n_{d}$ = 17,640)		0.35
**Sex **					
Male	26,388	51.85%	9713	55.06%	0.37
Female	24,508	48.15%	7927	44.94%	0.32
**Age, years**					
Mean age ± SD	68.71 ± 18.14		74.29 ± 15.23		-
18–39	4562	8.96%	577	3.27%	0.13
40–59	8999	17.68%	2206	12.51%	0.25
60–79	20,971	41.20%	7326	41.53%	0.35
≥80	16,364	32.15%	7531	42.69%	0.46
**Number of comorbidities**					
0	21,009	41.28%	6193	35.11%	0.29
1	16,721	32.85%	6010	34.07%	0.36
2	10,055	19.76%	4013	22.75%	0.40
3	2694	5.29%	1222	6.93%	0.45
≥4	417	0.82%	202	1.14%	0.48
**Vaccine doses**					
0–2 doses	40,657	79.88%	13,921	78.92%	0.34
≥3 doses	10,239	20.12%	3,719	21.08%	0.36

## Data Availability

The computational methodology was implemented in Python language using libraries provided by Scikit-learn: https://scikit-learn.org/stable/ (accessed on 10 May 2022). The public database cited in Section 2 is freely available at: https://opendatasus.saude.gov.br/dataset/srag-2021-e-2022 (accessed on 25 April 2022).
